# Subdiffusive Source Sensing by a Regional Detection Method

**DOI:** 10.3390/s19163504

**Published:** 2019-08-10

**Authors:** Weijing Song, Fudong Ge, YangQuan Chen

**Affiliations:** 1 School of Computer Science, China University of Geosciences, Wuhan 430074, China; 2 School of Engineering (MESA-Lab), University of California, Merced, CA 95343, USA

**Keywords:** source sensing, time fractional diffusion systems, regional detection method, strategic sensors, spy sensors

## Abstract

Motivated by the fact that the danger may increase if the source of pollution problem remains unknown, in this paper, we study the source sensing problem for subdiffusion processes governed by time fractional diffusion systems based on a limited number of sensor measurements. For this, we first give some preliminary notions such as source, detection and regional spy sensors, etc. Secondly, we investigate the characterizations of regional strategic sensors and regional spy sensors. A regional detection approach on how to solve the source sensing problem of the considered system is then presented by using the Hilbert uniqueness method (HUM). This is to identify the unknown source only in a subregion of the whole domain, which is easier to be implemented and could save a lot of energy resources. Numerical examples are finally included to test our results.

## 1. Introduction

Recently, the studies of transport dynamics in complex systems which exhibit the subdiffusion property have attracted increasing attention. Typical examples include the water in membranes for fuel cells [1], charge transport in amorphous semiconductors [2] or heating processes of the heterogeneous rod [3]. It is worth mentioning that the mean squared displacement of subdiffusion process is a power-law function of fractional exponent, which is smaller than that of the Gaussian diffusion process [4,5]. Due to the strong interactions between components in these processes, a rather complex dynamical behavior would emerge. Note that a fractional order derivative itself is a kind of convolution and naturally links to subdiffusion processes, time fractional diffusion system is confirmed in [5,6,7,8] to be used to efficiently describe these subdiffusion processes. Then, some model-based investigations are needed to deal with their rather complex dynamical behaviors.

Source seeking is a fundamental issue in nature and, currently, different approaches have been developed to study it for the non-fractional diffusion systems (see monographs [9,10] and the survey [11] for example). This is motivated by the fact that, in some practical applications, such as the pollution problems, the danger may increase if the source remains unknown [12]. However, from a practical point of view, engineers are more interested in the sensing problem that, if a source is detectable, how can it be identified based on a limited number of sensor measurements. Then, in this paper, we consider this source sensing problem for the subdiffusion processes governed by time fractional diffusion systems.

Motivated by these above considerations, in this paper, we deal with the following time fractional diffusion systems with a Riemann–Liouville fractional order derivative:

(1) $$\left\{\begin{array}{c} {}_{0}D_{t}^{\alpha }y\left(t\right)=Ay\left(t\right)+S\left(t\right),\;\;t\in \left[0,T\right],\;0<\alpha \leq 1,\\ \lim_{t\to 0^{+}}{}_{0}I_{t}^{1-\alpha }y\left(t\right)=y_{0}\in L^{2}\left(\Omega \right), \end{array}\right.$$

where $\Omega \subseteq R^{n}$ is an open bounded subset with a smooth boundary ∂Ω, ${}_{0}D_{t}^{\alpha }$ and ${}_{0}I_{t}^{\alpha }$ represent the Riemann–Liouville fractional order derivative and integral, respectively. Here, $S\left(t\right)$ denotes the unknown source to be specified later and *A* is the infinitesimal generator of a strongly continuous semigroup ${\left\{\Phi \left(t\right)\right\}}_{t\geq 0}$ in $L^{2}\left(\Omega \right)$. It is supposed that −A is a self-adjoint uniformly elliptic operator and, in addition, $y\in L^{2}\left(0,T;V\right)$, where *V* is a Hilbert space such that $V^{*}\subseteq L^{2}\left(\Omega \right)\subseteq V$ with continuous injections ($V^{*}$ is the dual of *V*).

It is worth noting that, although the initial condition for Riemann–Liouville type time fractional diffusion system does not take the same form as that of non-fractional differential equations, expressions like $\lim_{t\to 0^{+}}{}_{0}I_{t}^{1-\alpha }y\left(t\right)$ in system (1) make sense. The reason is that it does not require a direct experimental evaluation of these fractional integrals. Instead, one can get it by measuring the initial values of its “inseparable twin”, which is obtained based on some basic physical law for the particular field of science. That is, the physical meaning for the Riemann–Liouville fractional integral of a function is equivalent to the initial value of its “inseparable twin”. For example, in the fractional Voigt model (a spring and a spring-pot in parallel) of viscoelasticity, the physical meaning of a Riemann–Liouville fractional integral of the unknown strain ε(t) is in fact identical to the initial condition of its “inseparable twin”—the stress [13]. This is also consistent with the known fact that the spring in the Voigt model only affects long-term behavior. For more “inseparable twins”, we refer the reader to e.g., monographs [14,15] for more information on the pair of current and voltage in electrical circuits or the pair of temperature difference and heat flux in heat conduction, etc.

The applications of system (1) are rich in the real world. As stated in [16], system (1) is usually used to describe the dynamic process in spatially inhomogeneous environments. Typical examples include the flow through porous media with a source or sensing the source of groundwater flow, etc. The corresponding sensing techniques cited in this paper can also be used to enable more complex tasks such as landmine clearing, the disease spreading control in agriculture lands or the crowd evacuation in the case of emergencies.

Let the limited number of sensor measurements be given by

(2) $$\begin{array}{c} z(t)=Cy(t), \end{array}$$

where $C:L^{2}\left(0,T;V\right)\to L^{2}\left(0,T;Z\right)$ depends on the structure of sensors and *Z* is a Hilbert space. Then, the source sensing problem can be stated as follows:

Given the measurements $z\in L^{2}\left(0,T;Z\right)$, find a source *S* such that the solution of system (1) satisfies
  (3) $$\begin{array}{c} Cy(t)=z(t). \end{array}$$
  Several questions arise in such problems: can the available measurements *z* uniquely determine *S*? If so, how does *S* depend on *z* and is there an approach to determine it (sensing)?

In the past two decades, several numerical algorithm approaches have been proposed for the source sensing problem of non-fractional diffusion systems. In [17], fast algorithms to solve the source sensing problem for elliptic partial differential equations (PDEs) were presented, in which the solution was approximated by using the Fourier–Galerkin truncated method. By using the multidimensional frequency estimation techniques, a new framework for solving the source sensing problems for systems governed by linear PDEs was presented in [18,19]. In addition, if the source is assumed to be a sum of a finite number of Dirac delta functions at unknown locations, numerical algorithms for the source identification problem of linear heat equations and time-dependent advection-diffusion systems with a nonlinear reaction were considered in [20,21], respectively. For an overview of the optimisation approaches for pollution source sensing in groundwater, we refer the reader to [22] and the references cited therein, although it was confirmed in [23,24] that the transport phenomena under the ground should be a subdiffusion process governed by time fractional diffusion systems.

However, the investigations for the source sensing problem of time fractional diffusion systems are still very limited. This is due to the fact that there is a need for further studies on the optimization variant theory and gradient theory of fractional order systems. As a result, the above optimisation numerical methods seem to be inapplicable for system (1). Furthermore, a source detection method has been proposed by El Jai and Afifi [25], in which the source is characterized by three parameters according to its properties. Here, we adopt these concepts and introduce the notion of regional detection of unknown sources, where we are interested in the sensing of unknown source only in a subregion of the whole domain. As it will be shown, the idea of regional detection can surely save energy resources. In addition, it is easier to be implemented even for some cases where we have a possibility to detect it in the whole domain.

After the introduction, the mathematical concepts of source and detection are given in the next section. The third section is focused on the regional strategic sensors, regional spy sensors and their relationships. In Section 4, an approach on solving the source sensing problem is presented. Two applications are worked out in the end.

## 2. Preliminary Results

The purpose of this section is to introduce the notions of sources, detection and some basic results to be used thereafter.

### 2.1. Sources

Let I:=[0,T]. The definition of a source *S* is as follows:

**Definition** **1.** *[25] A source S is characterized by a triplet (Σ,g,I), where*

*1.*  *Σ(·):t∈I→Σ(t)⊆Ω represents the support of source that varies in time t;*
*2.*  *g(·,t):x∈Σ(t)→g(x,t) defines the intensity of source in x at time t;*
*3.*  *$I=\left\{t:g(\cdot ,t)\neq 0\;on\;\Sigma (t)\right\}$ denotes the support of g and represents the life duration of source S.*

Here, the support Σ(·), which describes the moving trajectory of the source, is usually determined by the evolution of some dynamic systems. With this, *S* is said to be a

- moving pointwise source if Σ(t) is reduced to a single point of Ω for all t∈I;
- moving zone source if Σ(t) is reduced to a region of Ω for all t∈I;
- boundary source if Σ(t)⊆∂Ω, t∈I and, in this case, we can define the similar pointwise/zone boundary sources;
- fixed source if Σ is independent of *t*, which may be pointwise, zone or boundary.

In addition, it is worth noting that, when discussing the sensing problem, the pointwise fixed source defined as Σ(t)={σ}⊆Ω, ∀t∈I is always used. In this case, Σ is independent of *t*, which is used to describe a single point of Ω.

### 2.2. Regional Detection

Since the detection of a source can be done by neglecting its life duration, we consider the source as a couple (Σ,g). Let the set of such sources be E. One has

(4) $$\begin{array}{c} E\subseteq F\left(0,T;P(\Omega )\right). \end{array}$$

Here, P(Ω) represents the set of parts of Ω and $F\left(0,T;*\right)$ denotes the space of functions f:I→∗. With this, E can be a vector space with convenient scalar product operations.

**Definition** **2.** *A source S is said to be detectable on I if the knowledge of system* (1)*, together with the output function* (2)*, is sufficient to guarantee that the operator*

(5) $$\begin{array}{c} Q:S\in E\to z\in L^{2}\left(0,T;Z\right) \end{array}$$

*is injective.*

However, in many cases, it is impossible or too costly to reconstruct all parameters of a source. Let ω be a non-empty, not necessarily connected subregion of Ω. In what follows, we introduce the concepts of regional detection.

Assume that the source is located in ω such that Σ(t)⊆ω,∀t∈I. Considering the subspace

(6) $$\begin{array}{c} E_{\omega }:=\left\{\left(\Sigma ,g\right)\in E:\Sigma \left(t\right)\subseteq \omega ,\;\forall t\in I,\;g\in L^{2}\left(0,T;L^{2}\left(\omega \right)\right)\right\} \end{array}$$

and defining the operator $Q_{\omega }:S\in E_{\omega }\to z\in L^{2}\left(0,T;Z\right),$ we obtain the following definition.

**Definition** **3.** *A source S is called to be ω−detectable on I if* (1), (2) *is sufficient to ensure that $Q_{\omega }$ is injective.*

Note that a source, which is $\omega_{1}-$detectable, is called to be $\omega_{2}-$detectable if $\omega_{1}\subseteq \omega_{2}\subseteq \Omega \;\mathrm{with}\;\Sigma \left(t\right)\subseteq \omega_{1}.$

### 2.3. Some Basic Results

To obtain our results, in this part, we present some basic results on fractional calculus.

**Definition** **4** ([26])**.** *The Riemann–Liouville fractional integral of order α>0 for a function y is given by*

(7) $$\begin{array}{c} {}_{0}I_{t}^{\alpha }y\left(t\right)=\int_{0}^{t}\frac{{\left(t-s\right)}^{\alpha -1}}{\Gamma (\alpha )}y\left(s\right)ds, \end{array}$$

*where Γ(α) represents the Euler gamma function defined by $\Gamma \left(\alpha \right)=\int_{0}^{\infty }t^{\alpha -1}e^{-t}dt$ and the right side is pointwise defined on [0,T].*

**Definition** **5** ([26])**.** *The Riemann–Liouville fractional derivative of order α∈(0,1] for a function y is defined as*

(8) $$\begin{array}{c} {}_{0}D_{t}^{\alpha }y\left(t\right)=\left\{\begin{array}{c} \frac{d}{dt}{}_{0}I_{t}^{1-\alpha }y\left(t\right),\;\alpha \in \left(0,1\right),\;\\ \frac{d}{dt}y\left(t\right),\;\;\alpha =1 \end{array}\right. \end{array}$$

*provided that the right side is pointwise defined on [0,T].*

Consider system (1); without loss of generality, suppose that y(t)≡0 and S(t)≡0 when t∉I.

Let

(9) $$\begin{array}{c} \tilde{y}\left(s\right)=\int_{-\infty }^{\infty }e^{-st}y\left(t\right)dt\;\mathrm{and}\;\tilde{S}\left(s\right)=\int_{-\infty }^{\infty }e^{-st}S\left(t\right)dt \end{array}$$

be the Laplace transforms of functions *y* and *S*. Based on

(10) $$\begin{array}{c} \begin{array}{c} L\;\left\{{}_{0}D_{t}^{\alpha }y\right\}\left(s\right)=sL\left\{{}_{0}I_{t}^{1-\alpha }y\right\}\left(s\right)-y_{0}=s^{\alpha }\tilde{y}\left(s\right)-y_{0},\;\alpha \in \left(0,1\right], \end{array} \end{array}$$

system (1) is equivalent to $s^{\alpha }\tilde{y}\left(s\right)-y_{0}=A\tilde{y}\left(s\right)+\tilde{S}\left(s\right),$ which yields

(11) $$\tilde{y}\left(s\right)={\left(s^{\alpha }I-A\right)}^{-1}\left(y_{0}+\tilde{S}\left(s\right)\right)=\int_{-\infty }^{\infty }e^{-s^{\alpha }\tau }\Phi \left(\tau \right)\left[y_{0}+\tilde{S}\left(s\right)\right]d\tau .$$

Then, if there exists a function $\psi_{\alpha }\left(t\right)$ such that its Laplace transform is

(12) $$\begin{array}{c} \int_{-\infty }^{\infty }e^{-st}\psi_{\alpha }\left(t\right)dt=e^{-s^{\alpha }},\;\;\alpha \in \left(0,1\right], \end{array}$$

let $\phi_{\alpha }\left(t\right)=\frac{1}{\alpha }t^{-1-\frac{1}{\alpha }}\psi_{\alpha }\left(t^{-\frac{1}{\alpha }}\right)$, El-Borai has shown in [27,28] that the unique solution of system (1) satisfies

(13) $$\begin{array}{c} \begin{array}{c} y\left(t\right)=\alpha \int_{0}^{\infty }\theta t^{\alpha -1}\phi_{\alpha }\left(\theta \right)\Phi \left(t^{\alpha }\theta \right)y_{0}d\theta +\alpha \int_{0}^{t}\int_{0}^{\infty }\theta {\left(t-\tau \right)}^{\alpha -1}\phi_{\alpha }\left(\theta \right)\Phi \left({\left(t-\tau \right)}^{\alpha }\theta \right)d\theta S\left(\tau \right)d\tau . \end{array} \end{array}$$

Here, $\psi_{\alpha }\left(t\right)$ can, for example, be [29],

(14) $$\begin{array}{c} \psi_{\alpha }\left(t\right)=\left\{\begin{array}{c} \frac{1}{\pi }\sum_{n=1}^{\infty }{\left(-1\right)}^{n-1}t^{-\alpha n-1}\frac{\Gamma (n\alpha +1)}{n!}\sin\left(n\pi \alpha \right),\;\;t\in \left(0,\infty \right),\\ 0,\;t\in (-\infty ,0]. \end{array}\right. \end{array}$$

In addition, for the sake of simplicity, let $K_{\alpha }\left(t\right)=\alpha \int_{0}^{\infty }\theta \phi_{\alpha }\left(\theta \right)\Phi \left(t^{\alpha }\theta \right)d\theta .$ Equation (13) yields that

(15) $$\begin{array}{c} \begin{array}{c} y\left(t\right)=t^{\alpha -1}K_{\alpha }\left(t\right)y_{0}+\int_{0}^{t}{\left(t-\tau \right)}^{\alpha -1}K_{\alpha }\left(t-\tau \right)S\left(\tau \right)d\tau . \end{array} \end{array}$$

For more knowledge on the expression of solutions to system (1), we refer the reader to [7,30,31] and the references cited therein.

## 3. Regional Strategic Sensors and Regional Spy Sensors

The aim of this section is to explore the notions of regional strategic sensors, regional spy sensors and their relationships.

### 3.1. Regional Strategic Sensors

Let $p_{\omega }:L^{2}\left(\Omega \right)\to L^{2}\left(\omega \right)$ be the projection operator in ω defined by $p_{\omega }{y=y|}_{\omega }$ and we use

(16) $$\begin{array}{c} p_{\omega }^{*}y\left(x\right):=\left\{\begin{array}{c} y(x),\;\;x\in \omega ,\\ 0,\;\;\;x\in \Omega \backslash \omega \end{array}\right. \end{array}$$

to denote its adjoint operator. Consider the following autonomous system:

(17) $$\left\{\begin{array}{c} {}_{0}D_{t}^{\alpha }y\left(t\right)=Ay\left(t\right),\;\;t\in I,\\ \lim_{t\to 0^{+}}{}_{0}I_{t}^{1-\alpha }y\left(t\right)=y_{0}\;\mathrm{supposed}\;\mathrm{to}\;\mathrm{be}\;\mathrm{unknown},\\ z(t)=Cy(t). \end{array}\right.$$

Based on (15), one has $z\left(t\right)=K\left(t\right)y_{0}:=Ct^{\alpha -1}K_{\alpha }\left(t\right)y_{0}.$

**Definition** **6.** *System (17) is said to be ω−weakly observable if*

(18) $$\begin{array}{c} Ker\left(K\left(t\right)p_{\omega }^{*}\right)=\left\{0\right\},\;\;t\in I. \end{array}$$

As pointed out in [32], a sensor can be described by a couple (D,f) such that D⊆Ω represents the support of the actuator and *f* denotes its spatial distribution. Then, to obtain our main results, it is supposed that the measurements are made by *p* sensors ${\left(D_{i},f_{i}\right)}_{1\leq i\leq p}$ and the output function becomes

(19) $$\begin{array}{c} z\left(t\right)={\left({\left(f_{1},y\left(t\right)\right)}_{L^{2}\left(D_{1}\right)},\cdots ,{\left(f_{p},y\left(t\right)\right)}_{L^{2}\left(D_{p}\right)}\right)}^{T},\;\;t\in I. \end{array}$$

Here, $L^{2}\left(\Omega \right)$ is a Hilbert space endowed with the inner product ${\left(\cdot ,\cdot \right)}_{L^{2}\left(\Omega \right)}$; *p* denotes the number of the sensors, $D_{i}\subseteq \Omega$ is the support of the sensors and $f_{i}\in L^{2}\left(\Omega \right)$ represents their spatial distributions. In this case, $Z=R^{p}$.

**Definition** **7.** *Sensors ${\left(D_{i},f_{i}\right)}_{1\leq i\leq p}$ are said to be ω−strategic if the system* (17) *is ω−weakly observable.*

For the self-adjoint uniformly elliptic operator −A with Dirichlet boundary conditions, i.e.,

(20) $$\begin{array}{c} D\left(A\right)=\left\{\xi \in L^{2}\left(\Omega \right):\xi \left(x\right)=0\;\mathrm{in}\;\partial \Omega \right\}, \end{array}$$

we see that the spectrum of (A,D(A)) is composed of eigenvalues and counting according to the multiplicities [33]. Then, there exists a sequence ${\left(\lambda_{j},\xi_{j}\right)}_{j\geq 1}$ such that

- $\lambda_{j}$ is real for each j=1,2,⋯, and $\lambda_{j}$ is the eigenvalue of *A* with multiplicities $r_{j}$ such that
  (21) $$\begin{array}{c} \begin{array}{c} 0>\lambda_{1}>\lambda_{2}>\cdots >\lambda_{j}>\cdots ,\;\;\lim_{j\to \infty }\lambda_{j}=-\infty . \end{array} \end{array}$$
- $\xi_{jk}\;\left(k=1,2,\cdots ,r_{j}\right)$, which is the non-trivial solution of the problem:
  (22) $$\begin{array}{c} \left\{\begin{array}{c} A\xi_{j}\left(x\right)=\lambda_{j}\xi_{j}\left(x\right),\;x\in \Omega ,\\ \xi_{j}\left(x\right)=0,\;x\in \partial \Omega , \end{array}\right. \end{array}$$
  is the eigenfunction corresponding to $\lambda_{j}$ such that ${\left(\xi_{jk_{m}},\xi_{jk_{n}}\right)}_{L^{2}\left(\Omega \right)}=\delta_{k_{m},k_{n}},$$k_{m},k_{n}=1,2,\cdots ,r_{j},$ where $\delta_{k_{m},k_{n}}$ is Kronecker delta function concentrated at the origin. In addition, we get that the sequence ${\left\{\xi_{jk}\right\}}_{j=1,2,\cdots ,k=1,2,\cdots ,r_{j}}$ forms a complete and orthonormal basis in $L^{2}\left(\Omega \right)$ and any $\varphi \in L^{2}\left(\Omega \right)$ can be expressed by
  (23) $$\begin{array}{c} \varphi \left(x\right)=\sum_{j=1}^{\infty }\sum_{k=1}^{r_{j}}\left(\varphi ,\xi_{jk}\right)\xi_{jk}\left(x\right). \end{array}$$

Note that the above assumptions on operator *A* is general. For example, if Ω=(0,1), $A=▵=\partial^{2}/\partial x^{2}$, then −A is a symmetric operator. Considering the Dirichlet boundary conditions z(0,t)=z(1,t)=0, we get that $\lambda_{n}=-n^{2}\pi^{2}$, $\xi_{n}\left(x\right)=\sqrt{2}\sin\left(n\pi x\right),\;n=1,2,\cdots$ and, in addition, ${\left\{\sqrt{2}\sin\left(n\pi x\right)\right\}}_{n\geq 1}$ forms a complete and orthonormal basis in $L^{2}\left(\Omega \right)$ [33].

We are now ready to state the following result.

**Theorem** **1.** *Define $p\times r_{j}$ matrices $G_{j}$ as*

(24) $$G_{j}={\left[\begin{array}{cccc} \xi_{j1}^{1} & \xi_{j2}^{1} & \cdots & \xi_{jr_{j}}^{1}\\ \xi_{j1}^{2} & \xi_{j2}^{2} & \cdots & \xi_{jr_{j}}^{2}\\ \vdots & \vdots & \vdots & \vdots \\ \xi_{j1}^{p} & \xi_{j2}^{p} & \cdots & \xi_{jr_{j}}^{p} \end{array}\right]}_{p\times r_{j}},$$

*where $\xi_{jk}^{i}={\left(\xi_{jk},f_{i}\right)}_{L^{2}\left(D_{i}\right)}$, i=1,2,⋯,p and $k=1,2,\cdots ,r_{j}$. Then, the sensors ${\left(D_{i},f_{i}\right)}_{1\leq i\leq p}$ are ω−strategic if and only if*

(25) $$\begin{array}{c} p\geq r=\max\left\{r_{j}\right\}\;and\;rank\;G_{j}=r_{j},\;\forall j=1,2,\cdots . \end{array}$$

**Proof.**  It follows from Definition 7 that the sensors ${\left(D_{i},f_{i}\right)}_{1\leq i\leq p}$ are ω−strategic if and only if

(26) $$\begin{array}{c} Ct^{\alpha -1}K_{\alpha }\left(t\right)p_{\omega }^{*}y=0\Rightarrow y=0,\;\forall y\in L^{2}\left(\omega \right). \end{array}$$

Considering that $\alpha E_{\alpha ,\beta }^{2}=E_{\alpha ,\beta -1}-\left(1+\alpha -\beta \right)E_{\alpha ,\beta }$ [7], where

(27) $$\begin{array}{c} E_{\alpha ,\beta }^{\mu }\left(z\right):=\sum_{n=0}^{\infty }\frac{{\left(\mu \right)}_{n}}{\Gamma (\alpha n+\beta )}\frac{z^{n}}{n!},z\in C,\alpha ,\beta ,\mu \in C,Re\left(\alpha \right)>0 \end{array}$$

is known as the generalized Mittag–Leffler function in three parameters. In particular, write $E_{\alpha ,\beta }^{0}\left(z\right)=E_{\alpha ,\beta }\left(z\right)$ and $E_{\alpha ,1}\left(z\right)=E_{\alpha }\left(z\right)$ for short when μ=0 and μ=0,β=1, respectively. With this, we have

(28) $$\begin{array}{c} \begin{array}{cc} K_{\alpha }\left(t\right)p_{\omega }^{*}y & =\alpha \int_{0}^{\infty }\theta \phi_{\alpha }\left(\theta \right)\Phi \left(t^{\alpha }\theta \right)p_{\omega }^{*}yd\theta \\ & =\alpha \int_{0}^{\infty }\theta \phi_{\alpha }\left(\theta \right)\sum_{j=1}^{\infty }\sum_{k=1}^{r_{j}}\exp\left(\lambda_{j}t^{\alpha }\theta \right)\left(p_{\omega }^{*}y,\xi_{jk}\right)\xi_{jk}\left(x\right)d\theta \\ & =\sum_{j=1}^{\infty }\sum_{k=1}^{r_{j}}\sum_{n=0}^{\infty }\frac{\alpha \left(n+1\right)!{\left(-\lambda_{j}t^{\alpha }\right)}^{n}}{\Gamma (\alpha n+\alpha +1)n!}\left(p_{\omega }^{*}y,\xi_{jk}\right)\xi_{jk}\left(x\right)\\ & =\sum_{j=1}^{\infty }\sum_{k=1}^{r_{j}}\alpha E_{\alpha ,\alpha +1}^{2}\left(\lambda_{j}t^{\alpha }\right)\left(p_{\omega }^{*}y,\xi_{jk}\right)\xi_{jk}\left(x\right)\\ & =\sum_{j=1}^{\infty }\sum_{k=1}^{r_{j}}E_{\alpha ,\alpha }\left(\lambda_{j}t^{\alpha }\right){\left(p_{\omega }^{*}y,\xi_{jk}\right)}_{L^{2}\left(\Omega \right)}\xi_{jk}, \end{array} \end{array}$$

which is following from the property $\int_{0}^{\infty }\theta^{\nu }\phi_{\alpha }\left(\theta \right)d\theta =\frac{\Gamma (1+\nu )}{\Gamma (1+\alpha \nu )}$ for some ν≥0 [29,30]. Consequently, the necessary and sufficient condition for strategic sensors ${\left(D_{i},f_{i}\right)}_{1\leq i\leq p}$ is that

(29) $$\begin{array}{c} \sum_{j=1}^{\infty }\sum_{k=1}^{r_{j}}\frac{E_{\alpha ,\alpha }\left(\lambda_{j}t^{\alpha }\right)}{t^{1-\alpha }}G_{j}{\left(p_{\omega }^{*}y,\xi_{jk}\right)}_{L^{2}\left(\Omega \right)}=\theta :=\left(0,\cdots ,0\right)\in R^{p}\Rightarrow y=0. \end{array}$$

Finally, we cover our proof by using Reductio ad Absurdum.

**Necessity.** If $p\geq r=\max\left\{r_{j}\right\}\;\;\mathrm{and}\;rank\;G_{j}<r_{j}\;\mathrm{for}\;\mathrm{some}\;j=1,2,\cdots ,$ there exists a non-zero vector ${\tilde{y}}_{j}={\left({\tilde{y}}_{j1},{\tilde{y}}_{j2},\cdots ,{\tilde{y}}_{jr_{j}}\right)}^{T}$ satisfying $G_{j}{\tilde{y}}_{j}=\theta .$ Then, we can construct a non-zero element $\tilde{y}\in L^{2}\left(\omega \right)$ with ${\tilde{y}}_{jk}=\left(p_{\omega }^{*}\tilde{y},\xi_{jk}\right)$, for which

(30) $$\begin{array}{c} Ct^{\alpha -1}K_{\alpha }\left(t\right)p_{\omega }^{*}\tilde{y}=\theta . \end{array}$$

This implies that the sensors ${\left(D_{i},f_{i}\right)}_{1\leq i\leq p}$ are not ω−strategic.

**Sufficiency.** If the sensors ${\left(D_{i},f_{i}\right)}_{1\leq i\leq p}$ are not strategic, we can find a element $\hat{y}\neq 0$, $\hat{y}\in L^{2}\left(\Omega \right)$ such that $Ct^{\alpha -1}K_{\alpha }\left(t\right)p_{\omega }^{*}\hat{y}=\theta .$ Since $E_{\alpha ,\alpha }\left(\lambda_{j}t^{\alpha }\right)/t^{1-\alpha }>0$ for all t≥0, there exists some $j^{*}=1,2,\cdots$ satisfying

(31) $$\begin{array}{c} \sum_{k=1}^{r_{j}}G_{j^{*}}{\left(p_{\omega }^{*}\hat{y},\xi_{j^{*}k}\right)}_{L^{2}\left(\Omega \right)}=\theta . \end{array}$$

Consequently, if $p\geq r=\max\{r_{j^{*}}\}$, it is sufficient to see that $rank\;G_{j^{*}}<r_{j^{*}}.$ The proof is complete. □

### 3.2. Regional Spy Sensors

Consider system (1) with measurements given by *p* sensors ${\left(D_{i},f_{i}\right)}_{1\leq i\leq p}$, we state the following definition of regional spy sensors, which may lead to numerous problems and pose challenging research topics at the same time.

**Definition** **8.** *Sensors are said to be ω−spy sensors if they can detect any unknown sources in $E_{\omega }\subseteq E$.*

### 3.3. The Relationships between ω−Spy Sensors and ω−Strategic Sensors

Note that the detection problem and the observation problem are different [34]. Consequently, it leads immediately to the difference between ω−strategic sensors and ω−spy sensors.

**Lemma** **1.** *Strategic (ω−strategic) sensors are spy (ω−spy) sensors, while the converse is not true.*

**Proof.**  Based on the conclusion in [35] that S→y(t) is injective but not surjective, it is not difficult to see that, if sensors are ω−strategic, they are ω−spy sensors, while the converse fails. Here, ω may be whole domain. The proof is finished. □

In addition, we explore the following further result. For the sake of convenience, it is assumed that $y_{0}=0$ in the following discussion by realizing that system (1) is linear.

**Theorem** **2.** *Suppose that g in S satisfying $g\in L^{2}\left(0,T;L^{2}\left(\omega \right)\right)$. Then, ${\left(D_{i},f_{i}\right)}_{1\leq i\leq p}$ are ω−spy sensors if and only if they are ω−strategic sensors.*

**Proof.**  From Lemma 1, strategic sensors are spy sensors. Then, we next focus on showing its converse.

For any unknown sources $S\in E_{\omega }\subseteq E$, define the operator $Q_{\omega }:E_{\omega }\to L^{2}\left(0,T;R^{p}\right)$ as

(32) $$\begin{array}{c} S\to \left(Q_{\omega }S\right)\left(t\right)=z\left(t\right)={\left(z_{1}\left(t\right),z_{2}\left(t\right),\cdots ,z_{p}\left(t\right)\right)}^{T}, \end{array}$$

where $\xi_{jk}^{i}={\left(\xi_{jk},f_{i}\right)}_{L^{2}\left(D_{i}\right)}$ and

(33) $$\begin{array}{c} z_{i}\left(t\right)=\sum_{j=1}^{\infty }\sum_{k=1}^{r_{j}}\int_{0}^{t}\frac{E_{\alpha ,\alpha }\left(\lambda_{j}{\left(t-\tau \right)}^{\alpha }\right)}{{\left(t-\tau \right)}^{1-\alpha }}{\left(S\left(\tau \right),\xi_{jk}\right)}_{L^{2}\left(\Omega \right)}d\tau \xi_{jk}^{i},\;i=1,2,\cdots ,p. \end{array}$$

Based on Definitions 3 and 8, the necessary and sufficient condition for the ω−spy sensors ${\left(D_{i},f_{i}\right)}_{1\leq i\leq p}$ is that $Q_{\omega }$ is injective. Then, if the sensors ${\left(D_{i},f_{i}\right)}_{1\leq i\leq p}$ are not ω−strategic, by Theorem 1, there exists an element $\hat{y}\neq 0,$$\hat{y}\in L^{2}\left(\omega \right)$ such that $\sum_{k=1}^{r_{j}}\xi_{j^{*}k}^{i}{\left(p_{\omega }^{*}\hat{y},\xi_{j^{*}k}\right)}_{L^{2}\left(\Omega \right)}=\theta$ for some $j^{*}=1,2,\cdots .$ That is,

(34) $$\begin{array}{c} Q_{\omega }p_{\omega }^{*}\hat{y}=\theta \;\mathrm{with}\;\hat{y}≢0. \end{array}$$

Therefore, since $S\in E_{\omega }$ and $g\in L^{2}\left(0,T;L^{2}\left(\omega \right)\right),$ let $\hat{g}=g+\hat{y}.$ One has

(35) $$\begin{array}{c} Q_{\omega }\hat{S}=Q_{\omega }S, \end{array}$$

where $\hat{S}$ is the source having $\hat{g}$ as its intensity. This means that $\hat{S}$ is not detectable. As a result, we conclude that ${\left(D_{i},f_{i}\right)}_{1\leq i\leq p}$ are not ω−spy sensors and the proof is finished. □

## 4. Source Sensing Approach

In this section, we show how to identify the source $S=\left(\Sigma ,g\right)\in E_{\omega }$ under the hypothesis that ${\left(D_{i},f_{i}\right)}_{1\leq i\leq p}$ are ω−spy sensors.

**Theorem** **3.** *If ${\left(D_{i},f_{i}\right)}_{1\leq i\leq p}$ are ω−spy sensors, then the source $S=\left(\Sigma ,g\right)\in E_{\omega }$ in system (1) can be uniquely identified by the observation z as the unique solution of the following equation*

(36) $$\begin{array}{c} \Lambda_{\omega }S=Q_{\omega }^{*}z. \end{array}$$

*That is, given any $S_{1},S_{2}\in E_{\omega }$, the equality $Q_{\omega }S_{1}=Q_{\omega }S_{2}$ could imply $S_{1}=S_{2}$.*

**Proof.**  Let $y_{i}\left(t\right),$i=1,2 be the solution of system

(37) $$\left\{\begin{array}{c} {}_{0}D_{t}^{\alpha }y_{i}\left(t\right)=Ay_{i}\left(t\right)+S_{i}\left(t\right),\;t\in I,\\ \lim_{t\to 0^{+}}{}_{0}I_{t}^{1-\alpha }y_{i}\left(t\right)=y_{0}. \end{array}\right.$$

Then, the difference $y\left(t\right):=y_{1}\left(t\right)-y_{2}\left(t\right)$ satisfies

(38) $$\left\{\begin{array}{c} {}_{0}D_{t}^{\alpha }y\left(t\right)=Ay\left(t\right)+S_{1}\left(t\right)-S_{2}\left(t\right),\;t\in I,\\ \lim_{t\to 0^{+}}{}_{0}I_{t}^{1-\alpha }y\left(t\right)=0. \end{array}\right.$$

In what follows, we divide the proof into three steps.

Step 1, we consider the following semi-norm

(39) $$\begin{array}{c} {∥S∥}_{F_{\omega }}={∥Q_{\omega }S∥}_{L^{2}\left(0,T;R^{p}\right)},\;S\in E_{\omega } \end{array}$$

and show that ${∥\cdot ∥}_{F_{\omega }}$ defines a norm for the space $F_{\omega }:=\bar{E_{\omega }}$. For this, we only need to prove that any $S\in E_{\omega }$ with ${∥S∥}_{F_{\omega }}=0$ could yield S=0 [36]. Indeed, by Definitions 3 and 8, since ${\left(D_{i},f_{i}\right)}_{1\leq i\leq p}$ are ω−spy sensors, we get that $Q_{\omega }$ is injective, i.e., $Q_{\omega }S=0$ could imply S=0. With this, we conclude that $F_{\omega }$ is a Hilbert space endowed with the norm ${∥\cdot ∥}_{F_{\omega }}$ and the inner product

(40) $$\begin{array}{c} {\left(S_{1},S_{2}\right)}_{F_{\omega }}:={\left(Q_{\omega }S_{1},Q_{\omega }S_{2}\right)}_{L^{2}\left(0,T;R^{p}\right)}. \end{array}$$

Step 2, we prove that the operator $\Lambda_{\omega }:F_{\omega }\to F_{\omega }^{*}$ given by

(41) $$\begin{array}{c} \begin{array}{c} \Lambda_{\omega }S=Q_{\omega }^{*}Q_{\omega }S \end{array} \end{array}$$

is an isomorphism from space $F_{\omega }$ into its dual $F_{\omega }^{*}.$ Here, $Q_{\omega }^{*}$ denotes the adjoint operator of $Q_{\omega }.$ Indeed, given any $v\in L^{2}\left(0,T;R^{p}\right)$, by (15), one has

(42) $$\begin{array}{c} \begin{array}{cc} {\langle Q_{\omega }S,v\rangle }_{L^{2}\left(0,T;R^{p}\right)\times L^{2}\left(0,T;R^{p}\right)} & ={\langle Cy(t),v\rangle }_{L^{2}\left(0,T;R^{p}\right)\times L^{2}\left(0,T;R^{p}\right)}\\ & =\int_{0}^{T}v\left(t\right)C\int_{0}^{t}{\left(t-\tau \right)}^{\alpha -1}K_{\alpha }\left(t-\tau \right)S\left(\tau \right)d\tau dt\\ & =\int_{0}^{T}\int_{\tau }^{T}v\left(t\right)C{\left(t-\tau \right)}^{\alpha -1}K_{\alpha }\left(t-\tau \right)dtS\left(\tau \right)d\tau . \end{array} \end{array}$$

Then, the duality relationship ${\langle Q_{\omega }S,v\rangle }_{L^{2}\left(0,T;R^{p}\right)\times L^{2}\left(0,T;R^{p}\right)}={\langle S,Q_{\omega }^{*}v\rangle }_{F_{\omega }\times F_{\omega }^{*}}$ and (28) yield that

(43) $$\begin{array}{c} \begin{array}{cc} \left(Q_{\omega }^{*}v\right)\left(t\right) & =\int_{t}^{T}{\left(\varsigma -t\right)}^{\alpha -1}K_{\alpha }\left(\varsigma -t\right)C^{*}v\left(\varsigma \right)d\varsigma \\ & =\sum_{j=1}^{\infty }\sum_{k=1}^{r_{j}}\int_{t}^{T}\frac{E_{\alpha ,\alpha }\left(\lambda_{j}{\left(\varsigma -t\right)}^{\alpha }\right)}{{\left(\varsigma -t\right)}^{1-\alpha }}{\left(C^{*}v\left(\varsigma \right),\xi_{jk}\right)}_{L^{2}\left(\Omega \right)}d\varsigma \xi_{jk}. \end{array} \end{array}$$

Based on (43), define

(44) $$\begin{array}{c} \begin{array}{c} \left(\Lambda_{\omega }S\right)\left(t\right):=\left(Q_{\omega }^{*}Q_{\omega }S\right)\left(t\right)=\sum_{j=1}^{\infty }\sum_{k=1}^{r_{j}}\int_{t}^{T}\left[\begin{array}{c} \frac{E_{\alpha ,\alpha }\left(\lambda_{j}{\left(\varsigma -t\right)}^{\alpha }\right)}{{\left(\varsigma -t\right)}^{1-\alpha }}\sum_{m=1}^{\infty }\sum_{n=1}^{r_{m}}\int_{0}^{\varsigma }\frac{E_{\alpha ,\alpha }\left(\lambda_{m}{\left(\varsigma -\tau \right)}^{\alpha }\right)}{{\left(\varsigma -\tau \right)}^{1-\alpha }}d\tau \times \\ {\left(S\left(\tau \right),\xi_{mn}\right)}_{L^{2}\left(\Omega \right)}{\left(C^{*}C\xi_{mn},\xi_{jk}\right)}_{L^{2}\left(\Omega \right)} \end{array}\right]d\varsigma \xi_{jk}. \end{array} \end{array}$$

It follows from (40) that

(45) $$\begin{array}{c} {\left(\Lambda_{\omega }S_{1},S_{2}\right)}_{L^{2}\left(0,T;R^{p}\right)}={\left(S_{1},S_{2}\right)}_{F_{\omega }}. \end{array}$$

Then, if we consider the linear mapping $\Lambda_{\omega }^{S_{1}}:F_{\omega }\to R$ given by

(46) $$\begin{array}{c} \Lambda_{\omega }^{S_{1}}S_{2}={\left(\Lambda_{\omega }S_{1},S_{2}\right)}_{L^{2}\left(0,T;R^{p}\right)}, \end{array}$$

it leads to $\left|\Lambda_{\omega }^{S_{1}}S_{2}\right|\leq ∥S_{1}{∥}_{F_{\omega }}{∥S_{2}∥}_{F_{\omega }}.$ Therefore, $\Lambda_{\omega }^{S_{1}}$ is a continuous operator and has a unique extension to $F_{\omega }$ such that

(47) $$\begin{array}{c} ∥\Lambda_{\omega }^{S_{1}}{∥}_{F_{\omega }^{*}}={∥S_{1}∥}_{F_{\omega }},\;\;\forall S_{1}\in F_{\omega }. \end{array}$$

Moreover, we obtain that the linear operator $\Lambda_{\omega }:F_{\omega }\to F_{\omega }^{*}$ is continuous. Then, $\Lambda_{\omega }$ is an isomorphism from $F_{\omega }$ to $F_{\omega }^{*}$, which is following from (45) and (47).

Step 3, based on Theorem 1.1 of [37], to complete the proof, we only need to show that $\Lambda_{\omega }$ is a coercive operator. That is, there exists a positive constant γ such that

(48) $$\begin{array}{c} {\langle \Lambda_{\omega }S,S\rangle }_{F_{\omega }\times F_{\omega }^{*}}\geq \gamma {∥S∥}_{F_{\omega }}^{2},\;\;\forall S\in F_{\omega }. \end{array}$$

In fact, with these above preliminaries, if $Q_{\omega }$ is injective, $F_{\omega }$ is Hilbert space endowed with the norm ${∥S∥}_{F_{\omega }}$ and the inner product

(49) $$\begin{array}{c} {\left(S_{1},S_{2}\right)}_{F_{\omega }}={\left(Q_{\omega }S_{1},Q_{\omega }S_{2}\right)}_{L^{2}\left(0,T;R^{p}\right)}. \end{array}$$

For any $S\in F_{\omega },$ one has

(50) $$\begin{array}{c} \begin{array}{c} {\langle \Lambda_{\omega }S,S\rangle }_{F_{\omega }\times F_{\omega }^{*}}={\langle Q_{\omega }^{*}Q_{\omega }S,S\rangle }_{F_{\omega }\times F_{\omega }^{*}}={\left(Q_{\omega }S,Q_{\omega }S\right)}_{L^{2}\left(0,T;R^{p}\right)}={∥S∥}_{F_{\omega }}. \end{array} \end{array}$$

Then, (36) has a unique solution. This means that any $S_{1},S_{2}\in E_{\omega }$ satisfying the equality $Q_{\omega }S_{1}=Q_{\omega }S_{2}$ could yield $S_{1}=S_{2}$. Consequently, the unknown source *S* is uniquely identified and the proof is finished. □

**Remark** **1.** *From Theorem 3, if ${\left(D_{i},f_{i}\right)}_{1\leq i\leq p}$ are ω−spy sensors, we get that the operator $Q_{\omega }$ is injective by using the knowledge of the considered time fractional diffusion system and the sensor measurements. The main tool used in the above proof is the duality theory and our results could be used for sensing both the bounded time-varying space-dependence source (the zone source) and the unbounded time-varying space-dependence source (the pointwise source). With this, we see that the obtained results can be considered as a generalization of that in [38]. In particular, if $y_{0}=0$ and S(t) in the integral (15) is uniformly bounded with respect to all t∈[0,T], i.e., |S(t)|≤M, ∀t∈[0,T] holds for some constant M>0, we have [7]*

(51) $$\begin{array}{c} \begin{array}{c} \left|y(t)\right|=\left|\int_{0}^{t}{\left(t-\tau \right)}^{\alpha -1}K_{\alpha }\left(t-\tau \right)S\left(\tau \right)d\tau \right|=\sum_{n=1}^{\infty }\int_{0}^{t}\frac{E_{\alpha ,\alpha }\left(\lambda_{n}{\left(t-\tau \right)}^{\alpha -1}\right)}{{\left(t-\tau \right)}^{1-\alpha }}\left|S(\tau )\right|d\tau \\ \;\leq M\sum_{n=1}^{\infty }\frac{1-E_{\alpha }\left(\lambda_{n}t^{\alpha -1}\right)}{\lambda_{n}}\leq M\sum_{n=1}^{\infty }\frac{1}{\lambda_{n}}, \end{array} \end{array}$$

*which is convergent and is consistent with the conditional stability results in Theorem 3.1 of [38].*

**Remark** **2.** *Note that Theorem 3 is obtained by assuming that the measurement doesn’t contain noise and the considered domain is regular so that the eigenvalue pairing of operator A satisfying Equations (21) and (22) is obtained. However, these assumptions may fail in some practical applications. For this, due to the memory effect of the fractional derivative, more new properties on fractional derivatives (or on Mittag–Leffler functions) and more constraints on a system operator are required. While interesting, we conclude that source sensing problems for time fractional diffusion systems under uncertain measurements of an irregular bounded domain and their robust analysis are of great interest.*

Next, we give a concrete algorithm to recover the unknown fixed zone source S=(ω,g(x)). Here, ω⊆Ω denotes the support of the source and $g\in L^{2}\left(\omega \right)$ represents its intensity.

Since ${\left\{\xi_{jk}\right\}}_{j\geq 1,k=1,2,\cdots ,r_{j}}$ forms a complete and orthonormal basis in $L^{2}\left(\Omega \right)$, $p_{\omega }^{*}g\in L^{2}\left(\Omega \right)$ can be rewritten as follows:

(52) $$\begin{array}{c} \begin{array}{c} p_{\omega }^{*}g\left(x\right)=\sum_{j=1}^{\infty }\sum_{k=1}^{r_{j}}g_{jk}\xi_{jk}\left(x\right). \end{array} \end{array}$$

Then, the source sensing problem is converted to identify the value of coefficients $g_{jk}.$ By (36), one has

(53) $$\begin{array}{c} \sum_{j=1}^{\infty }\sum_{k=1}^{r_{j}}g_{jk}\Lambda_{\omega }\xi_{jk}\left(x\right)=\left(Q_{\omega }^{*}z\right)\left(x\right). \end{array}$$

Multiplying both sides of (53) with $\xi_{mn}\left(x\right)$ yields that

(54) $$\begin{array}{c} \sum_{j=1}^{\infty }\sum_{k=1}^{r_{j}}{\left(\Lambda_{\omega }\xi_{jk},\xi_{mn}\right)}_{L^{2}\left(\Omega \right)}g_{jk}={\left(Q_{\omega }^{*}z,\xi_{mn}\right)}_{L^{2}\left(\Omega \right)},\forall m\geq 1,n=1,2,\cdots ,r_{m}. \end{array}$$

With this, set $D_{jk}^{mn}={\left(\Lambda_{\omega }\xi_{jk},\xi_{mn}\right)}_{L^{2}\left(\Omega \right)}$ and $F_{mn}={\left(Q_{\omega }^{*}z,\xi_{mn}\right)}_{L^{2}\left(\Omega \right)}$. For a big enough integer *J*, $g_{jk}$ can then be approximated by solving the following equation:

(55) $$\begin{array}{c} \sum_{j=1}^{J}\sum_{k=1}^{r_{j}}D_{jk}^{mn}g_{jk}=F_{mn},\;m=1,2,\cdots ,J,n=1,2,\cdots ,r_{m}. \end{array}$$

It is worth mentioning that the matrix of (55) is positive. Consequently, we have

(56) $$\begin{array}{c} \begin{array}{c} g\left(x\right)=p_{\omega }\sum_{j=1}^{J}\sum_{k=1}^{r_{j}}g_{jk}\xi_{jk}\left(x\right). \end{array} \end{array}$$

## 5. Further Remarks

Realize that the Caputo fractional order derivative is another widely used derivative in fractional order systems; in this section, we consider the source sensing problem for the following time fractional diffusion system with a Caputo fractional derivative:

(57) $$\left\{\begin{array}{c} {}_{0}^{C}D_{t}^{\alpha }y\left(t\right)=Ay\left(t\right)+S\left(t\right),\;\;t\in I,\;0<\alpha \leq 1,\\ y\left(0\right)=y_{0}, \end{array}\right.$$

where ${}_{0}^{C}D_{t}^{\alpha }y\left(t\right)=\left\{\begin{array}{c} {}_{0}I_{t}^{1-\alpha }\frac{d}{dt}y\left(t\right),\alpha \in \left(0,1\right),\;\\ \frac{d}{dt}y\left(t\right),\;\;\alpha =1 \end{array}\right.$ denotes the Caputo fractional derivative.

Taking a Laplace transform on both sides of system (57), it yields that

(58) $$\tilde{y}\left(s\right)=\frac{s^{\alpha -1}y_{0}+\tilde{S}\left(s\right)}{s^{\alpha }I-A}=\int_{-\infty }^{\infty }s^{\alpha -1}e^{-s^{\alpha }\tau }\Phi \left(\tau \right)y_{0}d\tau +\int_{-\infty }^{\infty }e^{-s^{\alpha }\tau }\Phi \left(\tau \right)\tilde{S}\left(s\right)d\tau .$$

Observing that $L\left\{{}_{0}I_{t}^{1-\alpha }y\right\}\left(s\right)=s^{\alpha -1}L\left\{y\right\}\left(s\right),\;\alpha \in \left(0,1\right]$ and

(59) $$\begin{array}{c} \begin{array}{c} {}_{0}I_{t}^{1-\alpha }\frac{E_{\alpha ,\alpha }\left(\lambda_{j}t^{\alpha }\right)}{t^{1-\alpha }}=\sum_{i=0}^{\infty }\int_{0}^{t}\frac{\lambda_{j}^{i}{\left(t-\tau \right)}^{-\alpha }\tau^{\alpha i+\alpha -1}}{\Gamma (1-\alpha )\Gamma (\alpha i+\alpha )}d\tau =\sum_{i=0}^{\infty }\frac{\lambda_{j}^{i}t^{\alpha i}}{\Gamma (\alpha i+1)}=E_{\alpha }\left(\lambda_{j}t^{\alpha }\right), \end{array} \end{array}$$

the unique solution of system (57) satisfies [7]

(60) $$\begin{array}{c} \begin{array}{cc} y\left(t\right) & ={}_{0}I_{t}^{1-\alpha }\left(t^{\alpha -1}K_{\alpha }\left(t\right)\right)y_{0}+\int_{0}^{t}{\left(t-\tau \right)}^{\alpha -1}K_{\alpha }\left(t-\tau \right)S\left(\tau \right)d\tau \\ & =\sum_{j=1}^{\infty }\sum_{k=1}^{r_{j}}E_{\alpha }\left(\lambda_{j}t^{\alpha }\right){\left(\xi_{jk},y_{0}\right)}_{L^{2}\left(\Omega \right)}\xi_{jk}+\sum_{j=1}^{\infty }\sum_{k=1}^{r_{j}}\int_{0}^{t}\frac{E_{\alpha ,\alpha }\left(\lambda_{j}{\left(t-\tau \right)}^{\alpha }\right)}{{\left(t-\tau \right)}^{1-\alpha }}{\left(S\left(\tau \right),\xi_{jk}\right)}_{L^{2}\left(\Omega \right)}d\tau \xi_{jk}. \end{array} \end{array}$$

For the approach on identifying the source $S\in E_{\omega }$ governed by system (57), however, the conclusions obtained in previous sections will never hold if the measurements are defined as in (19). This is due to the fact that $E_{\alpha }\left(\lambda_{j}t^{\alpha }\right)$ is usually not equal to $t^{\alpha -1}E_{\alpha ,\alpha }\left(\lambda_{j}t^{\alpha }\right)$ if α∈(0,1). Then, some new revised definition of the measurements should be introduced.

Observing that ${}_{0}D_{t}^{1-\alpha }E_{\alpha }\left(\lambda_{j}t^{\alpha }\right)=t^{\alpha -1}E_{\alpha ,\alpha }\left(\lambda_{j}t^{\alpha }\right)$ for any $\lambda_{j}\in R$, t≥0 following from (59), if the sensor measurements are revised to be given by *p* sensors ${\left(D_{i},f_{i}\right)}_{1\leq i\leq p}$ as follows:

(61) $$\begin{array}{c} \hat{z}\left(t\right)=C_{2}y\left(t\right):={\left({\left(f_{1},{}_{0}^{C}D_{t}^{1-\alpha }y\left(t\right)\right)}_{L^{2}\left(D_{1}\right)},\cdots ,{\left(f_{p},{}_{0}^{C}D_{t}^{1-\alpha }y\left(t\right)\right)}_{L^{2}\left(D_{p}\right)}\right)}^{T}. \end{array}$$

Consider system (57) with S=0, define $\hat{z}\left(t\right)=K_{2}\left(t\right)y_{0}:=C_{2}y\left(t\right)$, and we obtain the following result.

**Definition** **9.** System (57), (61) with S=0 is said ω−weakly observable if $Ker\left(K_{2}\left(t\right)p_{\omega }^{*}\right)=\left\{0\right\},\;t\in I.$

**Theorem** **4.** *Define $p\times r_{j}$ matrices $G_{j}$ as*

(62) $$G_{j}={\left[\begin{array}{cccc} \xi_{j1}^{1} & \xi_{j2}^{1} & \cdots & \xi_{jr_{j}}^{1}\\ \xi_{j1}^{2} & \xi_{j2}^{2} & \cdots & \xi_{jr_{j}}^{2}\\ \vdots & \vdots & \vdots & \vdots \\ \xi_{j1}^{p} & \xi_{j2}^{p} & \cdots & \xi_{jr_{j}}^{p} \end{array}\right]}_{p\times r_{j}},$$

*where $\xi_{jk}^{i}={\left(\xi_{jk},f_{i}\right)}_{L^{2}\left(D_{i}\right)}$, i=1,2,⋯,p and $k=1,2,\cdots ,r_{j}$. Then, the sensors ${\left(D_{i},f_{i}\right)}_{1\leq i\leq p}$ are ω−strategic for system (57) with S=0 if and only if*

(63) $$\begin{array}{c} p\geq r=\max\left\{r_{j}\right\}\;and\;rank\;G_{j}=r_{j},\;\forall j=1,2,\cdots . \end{array}$$

Since the proof of Theorem 4 is very similar to that of Theorem 1, we omit it.

Consider system (57), let the operator ${\hat{Q}}_{\omega }:E_{\omega }\to L^{2}\left(0,T;R^{p}\right)$ be given by

(64) $$\begin{array}{c} \left({\hat{Q}}_{\omega }S\right)\left(t\right)=\hat{z}\left(t\right)={\left({\hat{z}}_{1}\left(t\right),{\hat{z}}_{2}\left(t\right),\cdots ,{\hat{z}}_{p}\left(t\right)\right)}^{T}, \end{array}$$

where

(65) $$\begin{array}{c} {\hat{z}}_{i}\left(t\right):=\sum_{j=1}^{\infty }\sum_{k=1}^{r_{j}}\int_{0}^{t}\frac{E_{\alpha ,\alpha }\left(\lambda_{j}{\left(t-\tau \right)}^{\alpha }\right)}{{\left(t-\tau \right)}^{1-\alpha }}{\left(S\left(\tau \right),\xi_{jk}\right)}_{L^{2}\left(\Omega \right)}d\tau \xi_{jk}^{i},\;i=1,2,\cdots ,p. \end{array}$$

The source sensing problem is stated as follows:

Given the measurements $\hat{z}\in L^{2}\left(0,T;R^{p}\right)$ defined by (61), find a source *S* such that the solution of system (57) satisfies
  (66) $$\begin{array}{c} \left({\hat{Q}}_{\omega }S\right)\left(t\right)=\hat{z}\left(t\right). \end{array}$$

**Theorem** **5.** *Assume that ${\left(D_{i},f_{i}\right)}_{1\leq i\leq p}$ are ω−spy sensors. Then, the source $S=\left(\Sigma ,g\right)\in E_{\omega }$ in system (57) can be uniquely identified by the observation z in (61) as the unique solution of the following equation*

(67) $$\begin{array}{c} \Lambda_{\omega }S={\hat{Q}}_{\omega }^{*}z. \end{array}$$

**Proof.**  For any two solutions $y_{1}$, $y_{2}$ of system (57), their difference $y\left(t\right):=y_{1}\left(t\right)-y_{2}\left(t\right)$ satisfies

(68) $$\left\{\begin{array}{c} {}_{0}^{C}D_{t}^{\alpha }y\left(t\right)=Ay\left(t\right)+S_{1}\left(t\right)-S_{2}\left(t\right),\;t\in I,\\ y(0)=0. \end{array}\right.$$

Then, we divide the proof into three steps.

Step 1, if ${\left(D_{i},f_{i}\right)}_{1\leq i\leq p}$ are ω−spy sensors, we get that ${\hat{Q}}_{\omega }$ is injective. Then, the semi-norm

(69) $$\begin{array}{c} {∥S∥}_{F_{\omega }}={∥{\hat{Q}}_{\omega }S∥}_{L^{2}\left(0,T;R^{p}\right)},\;S\in E_{\omega } \end{array}$$

defines a norm for the space $F_{\omega }:=\bar{E_{\omega }}$. Therefore, $F_{\omega }$ is a Hilbert space endowed with the norm ${∥\cdot ∥}_{F_{\omega }}$ and the inner product ${\left(S_{1},S_{2}\right)}_{F_{\omega }}={\left({\hat{Q}}_{\omega }S_{1},{\hat{Q}}_{\omega }S_{2}\right)}_{L^{2}\left(0,T;R^{p}\right)}.$

Step 2, given any $v\in L^{2}\left(0,T;R^{p}\right)$, since

(70) $$\begin{array}{c} \begin{array}{c} {\langle {\hat{Q}}_{\omega }S,v\rangle }_{L^{2}\left(0,T;R^{p}\right)\times L^{2}\left(0,T;R^{p}\right)}=\int_{0}^{T}\sum_{j=1}^{\infty }\sum_{k=1}^{r_{j}}\int_{\tau }^{T}\frac{E_{\alpha ,\alpha }\left(\lambda_{j}{\left(t-\tau \right)}^{\alpha }\right)}{{\left(t-\tau \right)}^{1-\alpha }}{\left(C_{2}^{*}v\left(t\right),\xi_{jk}\right)}_{L^{2}\left(\Omega \right)}dt{\left(S\left(\tau \right),\xi_{jk}\right)}_{L^{2}\left(\Omega \right)}d\tau . \end{array} \end{array}$$

The duality relationship ${\langle {\hat{Q}}_{\omega }S,v\rangle }_{L^{2}\left(0,T;R^{p}\right)\times L^{2}\left(0,T;R^{p}\right)}={\langle S,{\hat{Q}}_{\omega }^{*}v\rangle }_{F_{\omega }\times F_{\omega }^{*}}$ leads to

(71) $$\begin{array}{c} \begin{array}{c} \left({\hat{Q}}_{\omega }^{*}v\right)\left(t\right)=\sum_{j=1}^{\infty }\sum_{k=1}^{r_{j}}\int_{t}^{T}\frac{E_{\alpha ,\alpha }\left(\lambda_{j}{\left(\varsigma -t\right)}^{\alpha }\right)}{{\left(\varsigma -t\right)}^{1-\alpha }}{\left(C_{2}^{*}v\left(\varsigma \right),\xi_{jk}\right)}_{L^{2}\left(\Omega \right)}d\varsigma \xi_{jk}. \end{array} \end{array}$$

Define ${\tilde{\Lambda }}_{\omega }:F_{\omega }\to F_{\omega }^{*}$ as

(72) $$\begin{array}{c} \begin{array}{cc} \left({\tilde{\Lambda }}_{\omega }S\right)\left(t\right) & :=\left(Q_{\omega }^{*}Q_{\omega }S\right)\left(t\right)\\ & =\sum_{j=1}^{\infty }\sum_{k=1}^{r_{j}}\int_{t}^{T}\left[\begin{array}{c} \frac{E_{\alpha ,\alpha }\left(\lambda_{j}{\left(\varsigma -t\right)}^{\alpha }\right)}{{\left(\varsigma -t\right)}^{1-\alpha }}\sum_{m=1}^{\infty }\sum_{n=1}^{r_{m}}\int_{0}^{\varsigma }\frac{E_{\alpha ,\alpha }\left(\lambda_{m}{\left(\varsigma -\tau \right)}^{\alpha }\right)}{{\left(\varsigma -\tau \right)}^{1-\alpha }}d\tau \times \\ {\left(S\left(\tau \right),\xi_{mn}\right)}_{L^{2}\left(\Omega \right)}{\left(C^{*}C\xi_{mn},\xi_{jk}\right)}_{L^{2}\left(\Omega \right)} \end{array}\right]d\varsigma \xi_{jk}. \end{array} \end{array}$$

Similar to Step 2 of Theorem 3, we get that the operator ${\tilde{\Lambda }}_{\omega }$ is an isomorphism from $F_{\omega }$ to its dual $F_{\omega }^{*}.$

Step 3, for any $S\in F_{\omega },$ one has

(73) $$\begin{array}{c} \begin{array}{c} {\langle {\tilde{\Lambda }}_{\omega }S,S\rangle }_{F_{\omega }\times F_{\omega }^{*}}={\langle {\hat{Q}}_{\omega }S,{\hat{Q}}_{\omega }S\rangle }_{L^{2}\left(0,T;R^{p}\right)\times L^{2}\left(0,T;R^{p}\right)}={∥S∥}_{F_{\omega }}. \end{array} \end{array}$$

Then, Theorem 1.1 of [37] yields that (67) has a unique solution. As a result, the unknown source *S* in system (57) is uniquely identified by the observation (61). The proof is finished. □

Similarly, for any unknown fixed zone source S=(ω,g(x)), by (52) and (67), we have

(74) $$\begin{array}{c} \sum_{j=1}^{\infty }\sum_{k=1}^{r_{j}}g_{jk}\Lambda_{\omega }\xi_{jk}\left(x\right)=\left({\hat{Q}}_{\omega }^{*}z\right)\left(x\right). \end{array}$$

For big enough integer *J*, then $g_{jk}$ can be approximated by multiplying both sides of (74) with $\xi_{mn}\left(x\right)$ as follows:

(75) $$\begin{array}{c} \sum_{j=1}^{J}\sum_{k=1}^{r_{j}}D_{jk}^{mn}g_{jk}={\hat{F}}_{mn},\;m=1,2,\cdots ,J,\;n=1,2,\cdots ,r_{m}, \end{array}$$

where $D_{jk}^{mn}={\left(\Lambda_{\omega }\xi_{jk},\xi_{mn}\right)}_{L^{2}\left(\Omega \right)}$ and ${\hat{F}}_{mn}={\left({\hat{Q}}_{\omega }^{*}z,\xi_{mn}\right)}_{L^{2}\left(\Omega \right)}$. With this, we obtain that

(76) $$\begin{array}{c} \begin{array}{c} g\left(x\right)=p_{\omega }\sum_{j=1}^{J}\sum_{k=1}^{r_{j}}g_{jk}\xi_{jk}\left(x\right). \end{array} \end{array}$$

## 6. Numerical Examples

The aim of this numerical work is to identify a fixed zone source $S\in E_{\omega }$ according to the methods given in Section 4.

Let $\Omega \subseteq R^{n}$ be an open bounded subset with smooth boundary ∂Ω, we consider the following system

(77) $$\begin{array}{c} \left\{\begin{array}{c} {}_{0}D_{t}^{\alpha }y\left(x,t\right)=▵y\left(x,t\right)+S\left(x,t\right)\;\mathrm{in}\;\Omega \times \left[0,T\right],\\ y(x,t)=0\;\mathrm{in}\;\partial \Omega \times [0,T],\\ \lim_{t\to 0^{+}}{}_{0}I_{t}^{1-\alpha }y\left(x,t\right)=0\;\mathrm{in}\;\Omega , \end{array}\right. \end{array}$$

where $▵=\partial^{2}/\partial x^{2}$ denotes the Laplace operator and $S\left(x,t\right)=\left(\omega ,g(x)\right)$ represents the unknown source to be sought. The measurements are made by *p* sensors ${\left(D_{i},f_{i}\right)}_{1\leq i\leq p}$ as follows:

(78) $$\begin{array}{c} z\left(t\right)=Cy\left(x,t\right)={\left({\left(f_{1},y\left(\cdot ,t\right)\right)}_{L^{2}\left(D_{1}\right)},\cdots ,{\left(f_{p},y\left(\cdot ,t\right)\right)}_{L^{2}\left(D_{p}\right)}\right)}^{T},\;\;t\in I. \end{array}$$

Based on the arguments in Section 4, the sensing problem of system (77) under measurements (78) can be solved via the following applicable steps:

1. Initial data α,Ω, *T* and the ω−spy sensors $\left(p,D_{i},f_{i}\right)$;
2. Given big enough integer *J*, obtain $D_{jk}^{mn}$ and $F_{mn}$ for all m=1,2,⋯J;
3. Solve the problem (55) to get $g_{jk}$ and then obtain *g* based on (52).

### 6.1. One-Dimensional Case

Let Ω=(0,1). We get that $\lambda_{j}=-j^{2}\pi^{2}$, $\xi_{j}\left(x\right)=\sqrt{2}\sin\left(j\pi x\right)$ and $r_{j}=1$. Here, ${\left\{\sqrt{2}\sin\left(j\pi x\right)\right\}}_{j\geq 1}$ forms a complete and orthonormal basis in $L^{2}\left(0,1\right)$[33]. Consider a fixed zone source S=(ω,g(x)) with the intensity *g* given by

(79) $$\begin{array}{c} g\left(x\right)=\left\{\begin{array}{c} (5.2-x)(x-0.4)+1,\;0.2\leq x<0.4;\\ 1,\;0.4\leq x<0.6;\\ (x-0.6)(x-5.8)+1,\;0.6\leq x<0.8;\\ 0,\;\mathrm{elsewhere}, \end{array}\right. \end{array}$$

where ω=[0.2,0.8] denotes the support of the source. Suppose that the measurements are made by one sensor (D,f). By Theorem 1, we obtain

**Proposition** **1.** *The sensor $\left(D,f\right)$ is ω−strategic for some subregion ω⊆Ω if and only if*

(80) $$\begin{array}{c} \int_{D}f\left(x\right)\sin\left(j\pi x\right)dx\neq 0 \end{array}$$

*holds true for all j=1,2,⋯.*

**Proof.**  Since $r_{j}=1,\;n=1$, it then follows that

(81) $$G_{1}=\sqrt{2}\int_{D}f\left(x\right)\sin\left(j\pi x\right)dx.$$

Theorem 1 shows that (80) holds and the proof is complete. □

In particular, if the support *D* of sensor reduces to a point δ(x−σ), we see that (80) is equal to σ∉Q. Without loss of generality, let the measurements be given by a pointwise sensor located at $\sigma =\sqrt{2}/3$ with the unit spatial distribution. Then, sensor (D,f) reduces to $\left(\delta \left(x-\sqrt{2}/3\right),1\right)$. With this, we get that

(82) $$\begin{array}{c} \begin{array}{cc} \left(Q_{\omega }g^{*}\right)\left(t\right) & =\sum_{j=1}^{\infty }\int_{0}^{t}{\left(t-\tau \right)}^{\alpha -1}E_{\alpha ,\alpha }\left(\lambda_{j}{\left(t-\tau \right)}^{\alpha }\right)d\tau {\left(g^{*},\xi_{j}\right)}_{L^{2}\left(\Omega \right)}\xi_{j}\left(\sigma \right)\\ & =\sum_{j=1}^{\infty }\frac{E_{\alpha }\left(\lambda_{j}t^{\alpha }\right)-1}{\lambda_{j}}{\left(g^{*},\xi_{j}\right)}_{L^{2}\left(\Omega \right)}\xi_{j}\left(\sigma \right), \end{array} \end{array}$$

which is injective. Therefore, $\left(Q_{\omega }^{*}z\right)\left(t\right)=\sum_{m=1}^{\infty }\int_{t}^{T}\frac{E_{\alpha ,\alpha }\left(\lambda_{m}{\left(\varsigma -t\right)}^{\alpha }\right)}{{\left(\varsigma -t\right)}^{1-\alpha }}z\left(\varsigma \right)d\varsigma \xi_{m}\left(\sigma \right)\xi_{m}$ and

(83) $$\begin{array}{c} \begin{array}{c} \left(\Lambda_{\omega }g^{*}\right)\left(t\right)=\sum_{m=1}^{\infty }\sum_{j=1}^{\infty }\int_{t}^{T}\frac{E_{\alpha ,\alpha }\left(\lambda_{m}{\left(\varsigma -t\right)}^{\alpha }\right)}{{\left(\varsigma -t\right)}^{1-\alpha }}\frac{E_{\alpha }\left(\lambda_{j}\varsigma^{\alpha }\right)-1}{\lambda_{j}}d\varsigma {\left(g^{*},\xi_{j}\right)}_{L^{2}\left(\Omega \right)}\xi_{j}\left(\sigma \right)\xi_{m}\left(\sigma \right)\xi_{m}. \end{array} \end{array}$$

Moreover, one has

(84) $$\begin{array}{c} \begin{array}{c} D_{j}^{m}={\left(\Lambda_{\omega }\xi_{j},\xi_{m}\right)}_{L^{2}\left(\Omega \right)}=\int_{t}^{T}\frac{E_{\alpha ,\alpha }\left(\lambda_{m}{\left(\varsigma -t\right)}^{\alpha }\right)}{{\left(\varsigma -t\right)}^{1-\alpha }}\frac{E_{\alpha }\left(\lambda_{j}\varsigma^{\alpha }\right)-1}{\lambda_{j}}d\varsigma \xi_{j}\left(\sigma \right)\xi_{m}\left(\sigma \right) \end{array} \end{array}$$

and

(85) $$\begin{array}{c} \begin{array}{c} F_{m}={\left(Q_{\omega }^{*}z,\xi_{m}\right)}_{L^{2}\left(\Omega \right)}=\int_{t}^{T}\frac{E_{\alpha ,\alpha }\left(\lambda_{m}{\left(\varsigma -t\right)}^{\alpha }\right)}{{\left(\varsigma -t\right)}^{1-\alpha }}z\left(\varsigma \right)d\varsigma \xi_{m}\left(\sigma \right). \end{array} \end{array}$$

**Figure 1:**
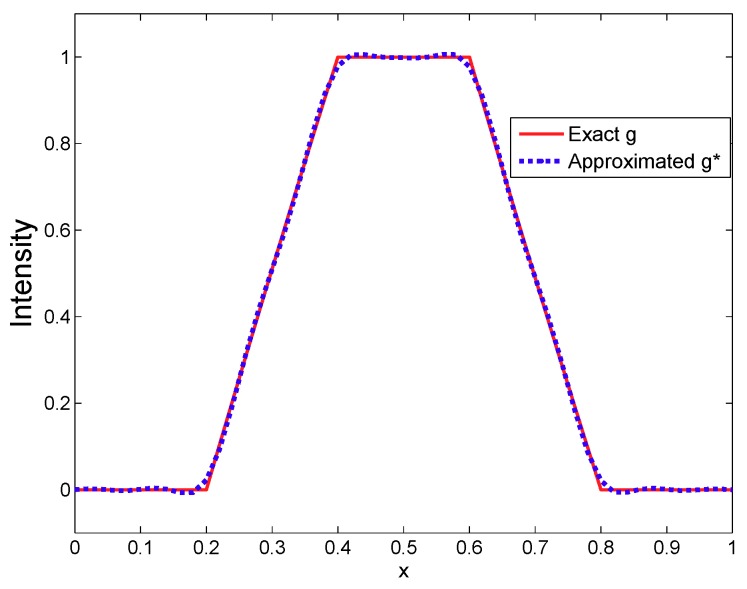
The exact intensity *g* and approximate intensity $g^{*}$ of the unknown fixed source.

Let α=0.5. Figure 1 shows how the approximated $g^{*}$ is close to *g*.

### 6.2. The Cases of n=2

This part focuses on the system (77) in $\Omega_{2}=\left(0,1\right)\times \left(0,1\right)\subseteq R^{2}$. In this case, the eigenvalues and corresponding eigenfunctions of operator *▵* are $\lambda_{ij}=-\left(i^{2}+j^{2}\right)\pi^{2}$ and $\xi_{ij}\left(x_{1},x_{2}\right)=2\sin\left(i\pi x_{1}\right)\sin\left(j\pi x_{2}\right)$, respectively. Here, $r_{ij}=1.$ Let the system (77) be excited by a fixed zone source $S=(\omega ,g\left(x_{1},x_{2}\right))$ with $g\left(x_{1},x_{2}\right)=\varphi \left(x_{1}\right)\psi \left(x_{2}\right)$ such that

(86) $$\begin{array}{c} \varphi \left(x_{1}\right)=\left\{\begin{array}{c} \left(5.2-x_{1}\right)\left(x_{1}-0.4\right)+1,\;0.2\leq x<0.4;\\ 1,\;0.4\leq x<0.6;\\ \left(x_{1}-0.6\right)\left(x_{1}-5.8\right)+1,\;0.6\leq x<0.8;\\ 0,\;\mathrm{elsewhere} \end{array}\right. \end{array}$$

and

(87) $$\begin{array}{c} \psi \left(x_{2}\right)=\left\{\begin{array}{c} \frac{25}{4}{\left(x_{2}-0.2\right)}^{2},\;0.2\leq x<0.6;\\ 25{\left(x_{2}-0.8\right)}^{2},\;0.6\leq x<0.8;\\ 0,\;\mathrm{elsewhere}. \end{array}\right. \end{array}$$

**Figure 2:**
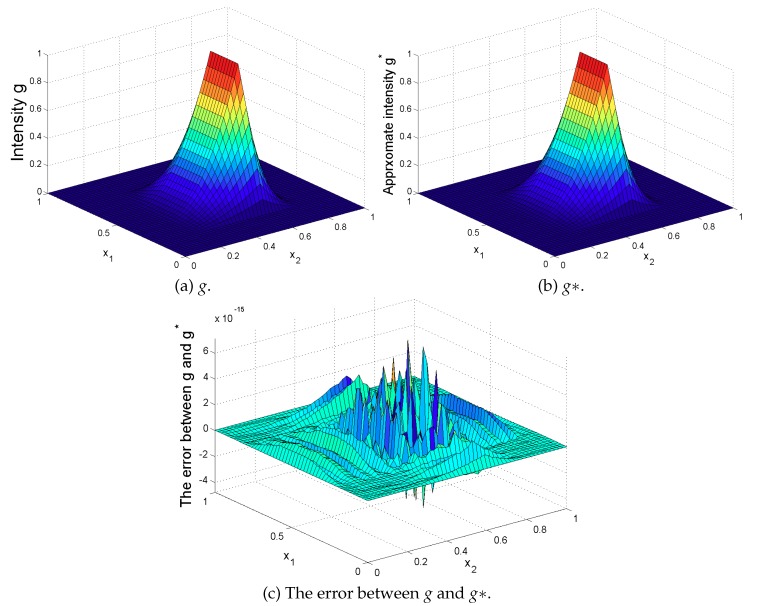
Comparison between the exact intensity *g* and the approximate intensity g*.

Then, the support of *S* is ω=[0.2,0.8]×[0.3,0.7]⊆Ω and its presentation is given by (a) of Figure 2.

Suppose that the measurements are made by one sensor (D,f). It follows that

**Proposition** **2.** *The sensor $\left(D,f\right)$ is ω−strategic for subregion $\omega \subseteq \Omega_{2}$ if and only if for all m,n=1,2,⋯,*

(88) $$\begin{array}{c} \int_{D}f\left(x_{1},x_{2}\right)\sin\left(m\pi x_{1}\right)\sin\left(n\pi x_{2}\right)dx_{1}dx_{2}\neq 0. \end{array}$$

The proof can be easily obtained similar to the proof of Proposition 1 following from Theorem 1. Then, we omit it.

Let $D=\left[\sqrt{2}/12,\sqrt{2}/6\right]\times \left\{0.5\right\}$ and f=1. Then, the sensor (D,f) reduces to $\left(\left[\sqrt{2}/12,\sqrt{2}/6\right]\times \left\{0.5\right\},1\right)$, which is a spy sensor. Moreover, it follows that $Q_{\omega }$ is injective and is defined as

(89) $$\begin{array}{c} \begin{array}{cc} \left(Q_{\omega }\hat{g}\right)\left(t\right) & =\sum_{i,j=1}^{\infty }\int_{0}^{t}{\left(t-\tau \right)}^{\alpha -1}E_{\alpha ,\alpha }\left(\lambda_{ij}{\left(t-\tau \right)}^{\alpha }\right)d\tau {\left(\hat{g},\xi_{ij}\right)}_{L^{2}\left(\Omega \right)}{\left(\xi_{ij},f\right)}_{L^{2}\left(D\right)}\\ & =\sum_{i,j=1}^{\infty }\frac{E_{\alpha }\left(\lambda_{ij}t^{\alpha }\right)-1}{\lambda_{ij}}{\left(\hat{g},\xi_{ij}\right)}_{L^{2}\left(\Omega \right)}{\left(\xi_{ij},f\right)}_{L^{2}\left(D\right)}. \end{array} \end{array}$$

Then, $\left(Q_{\omega }^{*}z\right)\left(t\right)=\sum_{m,n=1}^{\infty }\int_{t}^{T}\frac{E_{\alpha ,\alpha }\left(\lambda_{mn}{\left(\varsigma -t\right)}^{\alpha }\right)}{{\left(\varsigma -t\right)}^{1-\alpha }}z\left(\varsigma \right)d\varsigma {\left(\xi_{mn},f\right)}_{L^{2}\left(D\right)}\xi_{mn}$ and

(90) $$\begin{array}{c} \begin{array}{c} \left(\Lambda_{\omega }\hat{g}\right)\left(t\right)=\sum_{m,n=1}^{\infty }\sum_{i,j=1}^{\infty }\int_{t}^{T}\frac{E_{\alpha ,\alpha }\left(\lambda_{mn}{\left(\varsigma -t\right)}^{\alpha }\right)}{{\left(\varsigma -t\right)}^{1-\alpha }}\frac{E_{\alpha }\left(\lambda_{ij}\varsigma^{\alpha }\right)-1}{\lambda_{j}}d\varsigma {\left(\hat{g},\xi_{ij}\right)}_{L^{2}\left(\Omega \right)}{\left(\xi_{ij},f\right)}_{L^{2}\left(D\right)}{\left(\xi_{mn},f\right)}_{L^{2}\left(D\right)}\xi_{mn}. \end{array} \end{array}$$

Therefore, Theorem 3 shows that the source *S* can be sought from observation *z* by solving the equation $\Lambda_{\omega }S=Q_{\omega }^{*}z$. Let J=50. We get that

(91) $$\begin{array}{c} \begin{array}{c} D_{ij}^{mn}={\left(\Lambda_{\omega }\xi_{ij},\xi_{mn}\right)}_{L^{2}\left(\Omega \right)}=\int_{t}^{T}\frac{E_{\alpha ,\alpha }\left(\lambda_{mn}{\left(\varsigma -t\right)}^{\alpha }\right)}{{\left(\varsigma -t\right)}^{1-\alpha }}\frac{E_{\alpha }\left(\lambda_{ij}\varsigma^{\alpha }\right)-1}{\lambda_{j}}d\varsigma {\left(\xi_{ij},f\right)}_{L^{2}\left(D\right)}{\left(\xi_{mn},f\right)}_{L^{2}\left(D\right)} \end{array} \end{array}$$

and

(92) $$\begin{array}{c} \begin{array}{c} F_{mn}={\left(Q_{\omega }^{*}z,\xi_{mn}\right)}_{L^{2}\left(\Omega \right)}=\int_{t}^{T}\frac{E_{\alpha ,\alpha }\left(\lambda_{mn}{\left(\varsigma -t\right)}^{\alpha }\right)}{{\left(\varsigma -t\right)}^{1-\alpha }}z\left(\varsigma \right)d\varsigma {\left(\xi_{mn},f\right)}_{L^{2}\left(D\right)}. \end{array} \end{array}$$

Then, we refer the reader to (b) and (c) of Figure 2 on how close is the approximated $\hat{g}$ to the exact *g* when α=0.5.

## 7. Conclusions

The aim of this paper is to discuss the source sensing problem in a subdiffusion process by a regional detection method motivated by the great potential applications in environmental problems. The characterizations of regional strategic sensors, regional spy sensors and their relationships are presented. We discuss an approach on how to identify the unknown source only in a subregion of the whole domain by using the HUM. Some comparison results are given between time fractional diffusion system with a Riemann–Liouville fractional order derivative and that with a Caputo fractional order derivative. The results here can be regarded as an extension of the results in [25]. Moreover, we claim that some regularization method such as an iterative regularization method in [39] can be introduced to combine the concrete algorithm in this paper. Therefore, the source sensing problems for a fractional order distributed parameter systems by combining a regional detection method and the iterative regularization method as well as their comparisons with existing methods are of great interest.
